# The assessment of risk factors and risk perception status of breast cancer in Northern Iran

**DOI:** 10.1186/s12905-023-02422-z

**Published:** 2023-05-16

**Authors:** Reza Faryabi, Salman Daneshi, Esmat Rezabeigi Davarani, Ali Reza Yusefi, Mahdieh Arabpour, Khadijeh Ezoji, Ehsan Movahed, Milad Daneshi-Maskooni, Seyed Mohammadreza Hussaini

**Affiliations:** 1 School of Public Health, Jiroft University of Medical Sciences, Jiroft, Kerman Iran; 2 Health in Disasters and Emergencies Research Centre, Institute for Futures Studies in Health, Kerman University of Medical Sciences, Kerman, Iran; 3 School of Medicine, Jiroft University of Medical Sciences, Jiroft, Kerman Iran; 4 Social Determinants of Health Research Centre, Health Research Institute, Babol University of Medical Sciences, Babol, Iran; 5 Department of the Public Health, Faculty of Medical Sciences, Kateb University, Kabul, Afghanistan

**Keywords:** Risk factors, Breast cancer, Women, Risk perception, Northern Iran

## Abstract

**Background:**

Breast cancer (BC) is the most common malignancy in women. Identifying and avoiding the preventable risk factors of BC reduces its occurrence effectively. So, this study aimed to assess BC’s risk factors and risk perception status in Babol, Northern Iran.

**Methods:**

This cross-sectional study was conducted on 400 women aged 18 to 70 in Babol, Northern Iran. According to the eligibility criteria, the selected participants completed the demographic characteristics and researcher-made valid and reliable questionnaires. The statistical software was SPSS20.

**Results:**

The significant risk factors related to BC were old age (60 years old and more) (30.2%), obesity (25.8%), history of radiation (10%), and familial history of BC (9.5%), respectively (P < 0.05). Suspected symptoms of BC were observed in 78 (19.5%) women, including indentations in 27 (6.75%), redness in 15 (3.75%), pain in 16 (4%), and enlargement of lymph nodes in 20 (5%). The BC risk perception score was 107.72±13.22.

**Conclusion:**

Most participants had at least one risk factor for BC. It is essential to implement intervention programs to control obesity and BC screening programs in obese and overweight women to prevent BC and its complications. Further studies are needed.

## Introduction

Breast cancer (BC) is the most common cancer in women and the second leading cause of death from cancer [1]. In 2018, about 2.1 million new cases and 627,000 deaths of BC were reported worldwide [2]. The incidence of BC will increase worldwide by about 46% by 2046, according to the Global Cancer Observatory estimates [3]. The risk for BC in women’s lifetime is approximately one in eight worldwide [4]. BC ranks first among all cancers in women [5] and is the third most common cancer in Iran [6]. One in 10 to 15 Iranian women will likely develop BC [3]. About 7,000 to 9,000 new BC cases are identified annually in the country [7]. The onset of this cancer in Iran is earlier than in developed countries, and the most common age of onset is estimated at 35 to 45 years old [8].

BC is a multifactorial disease, and various factors such as ageing, premature puberty, late menopause, first pregnancy after the age of 30, infertility, no breastfeeding, genetic factors, chest radiography, birth control pills, socioeconomic status, smoking and alcohol consumption, inadequate physical activity, unhealthy diet, overweight and obesity, dense breast tissue, and a history of other cancers (especially ovarian and endometrial cancers) play a role in the progress of it [8–11]. BC is 100 times more common in women than men [4]. Due to lifestyle changes, BC has multiplied in most developing countries in recent decades [12]. More deaths from it have also been reported in less-developed areas [6]. BC grows slowly and can be diagnosed and treated in the early stages [13]. Early detection of BC prevents disease progression, increases patient survival, and reduces mortality [14, 15]. Studies have shown that high-risk perception influences the adoption of BC prevention behaviours and early disease detection [2, 16]. If the risk perception of disease increases, then protective measures may increase [17]. Understanding the disease risk is vital in performing preventive behaviours and adopting a healthy lifestyle.

Risk perception is a mental assessment and means knowing and making cognitive judgments about the nature and severity of a risk which varies from person to person [17]. If people do not find themselves at risk for the disease and underestimate the risk of the disease, they will not pay attention to the early signs of the disease. Moreover, low-risk perception can prevent preventive behaviours and lifestyle changes [18]. The mean of BC risk perception in participating women in Kerman was relatively low [6]. In a study in Nigeria, only 18% of rural women and 15% of urban women believed they might develop BC in their lifetime [19]. Cancers, especially breast cancer, are the most prevalent in women and need regular monitoring and measuring risk perception as an essential factor. It can cause the adoption of protective and preventive behaviours against breast cancer risk factors. Few studies have investigated the risk perception of breast cancer.

By identifying risk factors and lifestyle modifications, the morbidity and mortality of BC can be reduced. Identifying and avoiding known risk factors is the most straightforward and economical approach to preventing BC. Since no similar study has been done in Babol, this study was conducted to determine the prevalence of risk factors and the risk perception of BC in women aged 18 to 70 in northern Iran.

## Methods

This research is a cross-sectional (descriptive-analytical) study. The participants were women aged 18 to 70 living in Babol. The total female population of Babol city was 148,703 people, the studied population was 109,936 people, and the studied sample was 425 people.

**Fig. 1:**
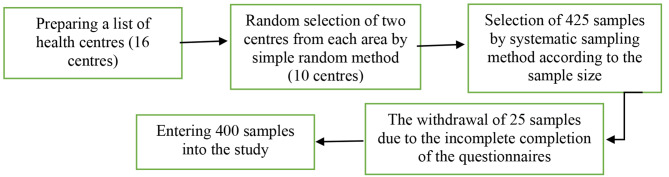
Flowchart of study

After obtaining the code of ethics and performing the necessary administrative coordination, 400 samples were included in the study by multi-stage sampling method. Thus, two health centres were selected from each part of the city (north, south, east, west, and centre). Samples were chosen according to the sample size and by a systematic sampling method in each centre. The following figure shows the flow chart of the multi-stage sampling process (Fig. 1).

After explaining the study’s objectives and obtaining informed verbal consent from participants before entering the study (by trained staff), the midwives performed a breast examination. Then anonymous questionnaires were completed by the study participants. The data collection tool in this study was a questionnaire containing demographic information (age, place of residence, marital status, education level, employment status, income level, number of children, and education in the case of Brest Cancer). It also included 32 questions related to BC risk factors and 33 questions related to the risk perception of BC, designed by the researcher. BC risk perception questions were assessed using a 5-point Likert scale from "strongly agree” to “strongly disagree” and in some questions from “absolutely” to “very much.“ To determine the risk perception status of the participants in the study, obtaining a score less than 50% of the possible score was considered poor, getting a score between 50 and 75% of the possible score was considered moderate, and receiving a score above 75% of the possible score was considered good [20].

Inclusion criteria were age 18–70 yrs, family records in health centres, no severe mental illness (diagnosed and entered in the registration systems of health care centres), and ability and willingness to participate. Based on the research objectives, a preliminary questionnaire was designed and prepared by studying books, articles, and scientific journals [21–24]. The questionnaire was provided to seven experts to determine the face validity and their opinions on clarity and transparency. Then the appearance of the questionnaire was reviewed and included in the final version. The questionnaire’s content validity was confirmed using relevant experts’ opinions on the content validity ratio and index. The content validity index score and the content validity ratio were 0.99 and 0.92, respectively. Thirty people were included in the study as a pilot to confirm the reliability. Cronbach’s alpha coefficient was 0.79. After completing the questionnaires, the data were entered into SPSS_20_. Quantitative variables were described using mean and standard deviation, including risk perception score, age, BMI [weight (kg)/ height (m)^2^], and physical inactivity (according to the WHO, the cut-point of less than 150 min of moderate activity per week (or equivalent)) [25]. Descriptive statistics were used to describe qualitative variables, including Residence, Marital status, Occupational status, Literacy level, Monthly income, Premature puberty, Late menopause, OCP, HRT, No breastfeeding, History of abortion, Radiation exposure, and Obesity. Inferential statistics were used to answer the research questions, including independent t-test and one-way variance analysis. A p-value < 0.05 was statistically significant.

## Results

The mean and standard deviation of the age of participants was 42.40 ± 17.41 years old. The majority of participants were urban residents (68.7%) and married (77.7%), and most participants in the study were a diploma or less (67.8%). Among married women, 78 (25.8%) had no children, 201 (64.63%) women had between one and three children, and 32 (10.28%) women had four or more children.

The mean and standard deviation of the BC risk perception score was 107.52 ± 13.22, with a range of 33 to 165 points possible. In other words, the participants obtained 65.16% of the risk perception score. In addition, the risk perception level was moderate. The employees had a higher risk perception than other jobs.

The relationship between the mean risk perception score and literacy level was significant (P < 0.001). It was higher in people with doctoral and master’s degrees compared to bachelor, diploma, undergraduate, and illiteracy.

Also, the mean risk perception score was significantly higher in married women than in single women (P = 0.03). Demographic characteristics and these relationships are presented in Table 1.

**Table 1:** The distribution of demographic characteristics and the relationship with risk perception of breast cancer

Variables		Number (Percent)	M ± SD of risk perception	P-value
**Residence**	**City**	275(68.7)	108.2 ± 14.8	0.49^*^
	**Village**	125(31.3)	107.2 ± 12.4	
**Marital status**	**Married**	89(22.3)	104.9 ± 10.5	0.03^*^
	**Single**	311(77.7)	108.2 ± 13.8	
**Occupational status**	**Self-employment**	131(32.8)	104.4 ± 9.1	0.06^**^
	**Student**	78(19.5)	105.3 ± 9.1	
	**Unemployed**	53 (13.3)	94.95 ± 11.55	
	**Staff**	103(25.8)	106.75 ± 11	
	**Housewife**	35(8.8)	103.8 ± 4.7	
**Literacy level**	**Illiterate**	82(20.5)	103.3 ± 4.1	0.0001>^**^
	**Under diploma**	94(23.5)	102.1 ± 3.6	
	**Diploma**	95(23.8)	102.8 ± 1.4	
	**Bachelor**	47(11.8)	108.2 ± 15.9	
	**Masters**	40(10)	120.1 ± 18.6	
	**Doctoral**	42(10.5)	124.9 ± 17.1	
**Monthly income level**	**Less than 46 dollars**	92(21.3)	107.1 ± 11.9	0.70^**^
	**Between 47 to 93**	110(27.5)	107.1 ± 12.9	
	**Between 94 and 186**	110(27.5)	108.7 ± 15.9	
	**Above 186 dollars**	88(22)	106.9 ± 10.8	

*T-test used to compare the means of two groups

**One-way ANOVA used to compare several populations means

A statistically significant relationship existed between the mean risk perception score and training history (P = 0.049). Suspected symptoms of BC were observed in 78 (19.5%) women. Breast indentations, redness of breasts, pain, enlargement of lymph nodes, and breast asymmetry were observed in 27 (6.75), 15 (3.75), 16 (4%), and 20 (5%), respectively. There was no statistically significant difference between the mean risk perception scores of women with clinical symptoms and other women (p = 0.387).

Among the women studied, 232 patients (58.2%) had a history of mammography. There was a statistically significant relationship between the mean risk perception score and history of mammography (p = 0.026). The most common risk factors associated with BC were old age (30.2%), obesity (25.8%), history of radiation (10%), and history of BC in the family and first-degree relatives (9.5%), respectively. None of the women in the study smoked.

A significant relationship was observed between the mean risk perception score with age > 45 yrs (P < 0.001), OCP^1^ (P = 0.043), infertility (P = 0.012), previous history of BC (P = 0.018), and radiation exposure (P = 0.044) (Table 2).

**Table 2:** The distribution of risk factors and the relationship with risk perception of breast cancer

Risk factors		N (%)	Mean ± SD^*^	P-value^**^	Risk factors		N (%)	Mean ± SD	P-value^**^
**Premature puberty**	**Yes**	27 (6.8)	104.59 ± 6.46	0.233	**Infertility**	**Yes**	23 (5.75)	110.52 ± 14.67	0.012
	**No**	373 (93.3)	107.73 ± 13.56			**No**	377 (94.2)	108.24 ± 13.93	
**Late menopause**	**Yes**	20 (5)	107.40 ± 12.62	0.966	**Get steroids**	**Yes**	197 (49.3)	106.54 ± 12.56	0.146
	**No**	380 (95)	107.52 ± 13.26			**No**	203 (50.8)	108.48 ± 13.80	
**OCP**	**Yes**	34 (5.5)	103.14 ± 5.22	0.043	**History of breast cancer**	**Yes**	2 (0.5)	120 ± 22.62	0.018
	**No**	366 (91.5)	107.93 ± 13.67			**No**	398 (99.5)	107.45 ± 13.17	
**HRT** 2	**Yes**	20 (5)	106.40 ± 11.64	0.697	**Contact with a smoker**	**Yes**	218 (54.5)	107.78 ± 13.96	0.666
	**No**	380 (95)	107.58 ± 13.31			**No**	182 (45.5)	107.20 ± 12.32	
**No breastfeeding**	**Yes**	98 (31.5)	106.65 ± 12.95	0.320	**Hookah consumption**	**Yes**	15 (3.8)	110.14 ± 14.37	0.051
	**No**	135 (43.4)	109.05 ± 16.06			**No**	385 (96.2)	107.42 ± 13.18	
**History of abortion**	**Yes**	13 (4.2)	107 ± 11.71	0.711	**Physical inactivity**	**Yes**	368 (92)	107.53 ± 13.31	0.620
	**No**	299 (95.8)	108.46 ± 13.95			**No**	32 (8)	107.38 ± 12.27	
**Radiation exposure**	**Yes**	40 (10)	103.52 ± 7.95	0.044	**Death of close relatives due to breast cancer**	**Yes**	11 (2.8)	104.45 ± 7.18	0.132
	**No**	360 (90)	107.97 ± 13.63			**No**	389 (97.2)	107.84 ± 13.66	
**Obesity (BMI over 30)**	**Yes**	103 (25.7)	106.16 ± 13.66	0.229	**Age over 45 years**	**Yes**	234 (58.5)	106.48 ± 11.54	0.000
	**No**	297 (74.3)	106 ± 13.05			**No**	166 (41.5)	108.96 ± 15.17	

*Mean and Standard deviation of risk perception

**t-test used to compare the means of two groups

The results showed that 66% of women had no risk factors for breast cancer. Most women over 50 had more than two risk factors (Table 3).

**Table 3:** Population distribution based on the number of exposure to risk factors by age groups

Age groups	Examined Subjects N (%)	No exposure to the risk factor (%)	One risk factor N (%)	Two risk factors N (%)	Three risk factors N (%)	Four risk factors and more N (%)
20–30 years	95 (23.8)	80 (84.2)	10 (10.5)	4 (4.2)	1 (1.05)	0 (0)
30–40 years	154 (38.5)	130 (84.4)	15 (9.7)	6 (3.9)	3 (1.9)	0 (0)
40–50 years	98 (24.5)	54 (55.1)	25 (25.5)	15 (15.3)	4 (4.08)	0 (0)
50–60 years	30 (7.5)	0 (0)	0 (0)	17 (56.7)	10 (33.3)	3 (10)
60–70 years	23 (5.7)	0 (0)	0(0)	7 (30.4)	13 (56.5)	3 (13.04)
Sum	400 (100)	264 (66)	50 (12.5)	49 (12.3)	31 (7.5)	6 (1.5)

## Discussion

This study aimed to determine the prevalence of risk factors and the perception of BC risk in women aged 18 to 70 years old in Babol. This study showed that 33.8% of the women had one or more risk factors for BC. In the study of Badrian et al., 78.79% of the participants had at least one risk factor for BC [26]. In the study of Rohparvarzadeh et al., 61.5% of women were not exposed to any risk factors, and 20% had at least one risk factor [27]. As the prevalence of BC is not the same in different regions, each country can have its risk factors, and its prevalence is different in other areas..

The study showed that in terms of the frequency of risk factors in the investigated women, the most common risk factors related to BC, respectively from the highest to the lowest frequency, were old age, obesity, history of radiation and history of BC in the family and first-degree relatives.

The prevalence of BC in similar studies in Ghana, Turkey and Iran (Isfahan) differed. In addition, the common risk factors, especially a family history of breast cancer (9.5 to 58.7%) and physical activity (8 to 64%), were dissimilar [2, 26, 28].

Studies have shown a significant relationship between age and BC risk, and increasing age has been introduced as one of the most important risk factors for BC [4, 10, 29]. After old age, obesity was the most common risk factor for BC in the present study. In the study of Rohparvarzadeh in Isfahan and Badrian et al. in Dehaghan, a body mass index of 30 and more was introduced as one of the main risk factors for BC [26, 27]. In a study by Yüksel et al., Which examined the risk factors for BC in Turkish women, 40.5% of participants were overweight, and 31.8% were obese [28].

A study showed that being overweight and obese increases the risk of developing BC [30]. Lifestyle changes such as inadequate physical activity and a sedentary lifestyle, as well as unhealthy eating patterns, have led to the prevalence of obesity, which is a significant risk factor for BC [10]. According to the program of care for risk factors for non-communicable diseases, the prevalence of obesity and overweight among Iranian women was 29.77% and 34.96%, respectively [31]. A case-control study in Arak showed that improper eating patterns increase the risk of BC [29]. In studies to determine the prevalence of BC risk factors, including Osei et al.‘s study, 36% of the students did not have regular physical activity [2]. In Rohparvarzadeh et al. and Badrian et al., irregular physical activity and inactivity were the most common risk factors for BC [26, 27]. History of radiation was the third highest risk factor identified in the present study, and 10% of participants had a history of radiation. Numerous studies have shown that moderate to high doses of radiation is a risk factor for BC [32]. In a study in Mashhad, 68.8% of breast cancer patients had a history of radiation, while in the control group, this rate was 53.1% [33].

In this study, 9.5% of the women reported a history of BC in the family and first-degree relatives. In the study of Osei et al., 14.3% of participants had a family history of BC [2]. In studies, a history of BC in relatives is one of the important risk factors for BC; it is known in women [5, 10, 29]. A family history of BC has been reported in almost a quarter of all breast cancers [4]. The results of the present study showed that the mean score of BC risk perception was moderate (65.16% of the 100% score). In a survey by Yavan et al., in Turkey, about 50% of women believed they were less likely to develop BC [34]. In a study by Osei et al., in Ghana, the risk perception score in medical students was 54.8% [2]. The possible reasons for the higher risk perception score in the present study may be the different participants (women versus students), referral of women to health centres for care and training (which may have a positive effect on their risk perception score), and the students being younger (mostly visit fewer health centres and consequently receive less training about breast cancer).

In this study, 9.5% of the women reported a history of BC in the family and first-degree relatives. In the study of Osei et al., 14.3% of participants had a family history of BC [2]. In studies, a history of BC in relatives is one of the important risk factors for BC; it is known in women [5, 10, 29]. A family history of BC has been reported in almost a quarter of all breast cancers [4]. The results of the present study showed that the mean score of BC risk perception was moderate (65.16% of the 100% score). In a survey by Yavan et al., in Turkey, about 50% of women believed they were less likely to develop BC [34]. In a study by Osei et al., in Ghana, the risk perception score in medical students was 54.8% [2]. The possible reasons for the higher risk perception score in the present study may be the different participants (women versus students), referral of women to health centres for care and training (which may have a positive effect on their risk perception score), and the students being younger (mostly visit fewer health centres and consequently receive less training about breast cancer).

In a study by Haji on women aged 35 to 85 years in Babol, the results showed that the perceived risk of breast cancer was significantly higher than the actual risk [1].

In the present study, people with higher literacy levels had a higher mean risk perception. In similar studies, with increasing education levels, the study group’s knowledge, risk perception, and adoption of preventive behaviours increased [1, 2, 6, 11, 14, 29, 34, 35]. Educated persons are probably more likely than others to seek reliable information about diseases, including BC. Increasing knowledge of risk factors, symptoms, incidence, and mortality can affect risk perception and preventive behaviours. The present study showed that the risk perception level in married women was higher than in single women. In the study of Khaleghi Nejad et al., in Kerman, married people had a higher self-test score than single people [36]. The consequences of morbidity or mortality of BC in married women can seriously affect various aspects of children and spouses’ physical and mental health and even the educational and work future of children and their spouses. Therefore, they pay more attention to their health because of it and would like to increase their awareness and understanding of the risk factors for this complication.

In the present study, women who had previously had a mammogram had a higher average perception of disease risk. In the study by Osei et al., students who were screened for breast cancer or planned to have it in the future were more likely to be at risk [2]. In their study, Yavan et al. reported that increased risk perception was associated with increased regular breast cancer screening [34]. While in the study of Zahedi et al., risk perception did not directly affect mammography [6]. Although increased risk perception of disease sometimes leads to behaviours such as mammography, one of the barriers to mammography is the fear of getting breast cancer [37]. In this study, the mean score of perception of BC risk in women taking birth control pills was significantly lower than in other women. Women more aware of the risk of BC are more likely to be cautious about taking hormonal medications. Contrary to the results of the present study, in the study of Osei et al., students who took oral contraceptive pills had a higher risk perception of BC [2]. The women in our study may only be thought that contraceptive pills prevent breast cancer, so their risk perception was low. However, in the study conducted on medical students taking contraceptive pills, the risk perception of breast cancer was higher than the women in the present study, probably because of studying about the side effects of them.

Therefore, women’s counselling is essential in health care centres and private offices, especially in high-risk cases using contraceptives. In this study, women who reported a previous history of BC had a higher risk of BC. In a South Korean study, women with a history of benign breast disease and a family history of BC were at greater risk [41]. In Haber et al.‘s study, the perception of BC risk was associated with first-degree relatives, and a family history of breast cancer influenced recurrent mammographic behaviour [38]. In the study of Osei et al., Medical students with no family history of BC were 90% less likely to develop BC than those who reported a family history of BC [2].

In a study in Brazil, women without a family history of BC had a lower risk of developing the disease and were less likely to have mammography [35]. Women with a history of morbidity in themselves or close relatives are more likely to know about the disease. Having more information and their suffering from this disease has led to a greater understanding of their danger. In the present study, women who reported a history of radiation exposure had a significantly lower risk perception than other women. In extreme cases, perceptions of high risk in some women may be a barrier to receiving radiation [39, 40]. Also, in this study, the mean risk perception in infertile women was significantly higher than in other women. Infertile women may seek information about their problem and its complications due to infertility, which curiosity and extensive study may increase their knowledge and understanding of the risk of BC. The present study showed that 60 to 70 years old had the highest exposure to BC risk factors which can be explained by the fact that older person has more risk factors, which probably increases the chances of developing BC with age.

### Strengths and limitations

Since breast cancer ranks first among cancers in women, emphasizing risk perception and assessing risk factors can help women increase their sensitivity to protective measures by identifying the main risk factors. However, this study had limitations such as using a self-report questionnaire, cross-sectional nature, and small sample size, so we should be careful to generalize the results.

## Conclusion

The most present study’s women had at least one risk factor for BC. Obesity was the current study’s most common modifiable risk factor for BC. The mean score of perception of BC risk was moderate. There was a statistically significant relationship between risk perception score and education, marital status, use of birth control pills, history of radiation, infertility, and history of BC. It is essential to implement intervention programs to control obesity and BC screening programs in obese and overweight women to prevent BC and its complications.

Given that the study is first, it is suggested to be studied further research with a higher sample size and period (mainly qualitative studies).

## Data Availability

The datasets used or analyzed during the current study are available from the corresponding author on a reasonable request.

## Footnotes

1 Oral Contraceptive Pills.

2 Hormone Replacement Therapy.
